# Supplementary material for: Longitudinal Predictors of Coronavirus-Related PTSD among Young Adults from Poland, Germany, Slovenia, and Israel
Source: Int J Environ Res Public Health. 2022 Jun 12;19(12):7207. doi: 10.3390/ijerph19127207 (PMC9222994; doi:10.3390/ijerph19127207)
Supplement: Supplementary file 1 [file ijerph-19-07207-s001.zip › ijerph-1754353-supplementary.pdf]

Table S1. Demographic characteristics of the study sample.

Demographic Variables	Total		Poland		Germany		Slovenia		Israel	
	n	%	*n*	%	*n*	%	*n*	%	*n*	%
Gender										
Women	935	54.30	222	49.80	224	53.60	247	57.30	242	56.50
Men	782	45.40	221	49.60	193	46.20	183	42.50	185	43.20
Did not want to say	6	0.30	3	0.70	1	0.20	1	0.20	1	0.20
Age										
20–30 years	840	48.80	236	52.90	202	48.30	202	46.90	200	46.70
31–40 years	883	51.20	210	47.10	216	51.70	229	53.10	228	53.30
Place of residence										
Village	426	24.70	155	34.80	71	17.00	162	37.60	28	8.90
Town (under 20,000 inhabitants)	310	18.00	63	14.10	84	20.10	120	27.80	43	10.00
City (20,000–99,000 inhabitants)	368	21.40	82	18.40	98	23.40	62	14.40	126	29.40
City (100,000–500,000 inhabitants)	380	22.10	85	19.10	82	19.60	65	15.10	148	34.60
Agglomeration (over 500,000 inhabitants)	239	19.90	61	13.70	83	19.90	22	5.10	73	17.10
Employment status										
Employed	1227	71.20	324	72.60	304	72.70	284	65.90	315	73.60
Unemployed	399	23.20	93	20.90	91	21.80	123	28.50	92	21.50
Self-employed	97	5.60	29	6.50	23	5.50	24	5.60	21	4.90
Total	1723	100	446	25.90	418	24.30	431	25.00	428	24.80
